# *Trimma charon*, a new species of pygmygoby (Teleostei, Gobiidae) from the Red Sea with a key to the Red Sea species of the genus

**DOI:** 10.3897/zookeys.1293.204097

**Published:** 2026-09-24

**Authors:** Viktor N. Peinemann, Sergey V. Bogorodsky, Matthew D. Tietbohl Talas, Michael L. Berumen

**Affiliations:** 1 Marine Science Program, Division of Biological and Environmental Science and Engineering, King Abdullah University of Science and Technology, Thuwal 23955, Saudi Arabia Marine Science Program, Division of Biological and Environmental Science and Engineering, King Abdullah University of Science and Technology Thuwal Saudi Arabia; 2 Senckenberg Research Institute and Natural History Museum Frankfurt, Senckenberganlage 25, 60325 Frankfurt am Main, Germany Senckenberg Research Institute and Natural History Museum Frankfurt Frankfurt am Main Germany

**Keywords:** Biodiversity, coral reef, cryptobenthic, endemism, identification key, Indian Ocean, mesophotic, taxonomy

## Abstract

*Trimma charon***sp. nov**. is described based on two specimens collected at 30 m depth from the northern Red Sea, Saudi Arabia. The new species can be distinguished from its congeners by the following combination of characters: predorsal midline with scales; fifth pelvic-fin ray unbranched and 41–43% length of the fourth ray; cheek and opercle scaleless; 14 pectoral-fin rays, all unbranched; dorsal fins VI + I,8, with no elongate spines; anal-fin I,8; bony interorbital width 55–62% of pupil diameter; and 6 papillae in row c. In life, *T. charon***sp. nov**. is translucent with a distinct, broad, orange-red midlateral stripe extending from the eye to the caudal-fin base and interrupted by three narrow pale bars, and with conspicuous golden spots on the caudal fin that extend onto the caudal peduncle; the iris is divided into four uneven white/orange sectors. The species is known from two specimens collected at the entrance of a cave on a steep outer-reef slope. *Trimma charon***sp. nov**. was compared with congeners, especially with the very similar *T. anaima* and co-occurring Red Sea species. An illustrated dichotomous key to all *Trimma* currently known from the Red Sea is provided. With the description of *T. charon***sp. nov**., the number of valid species in the Red Sea rises to 11.

## Introduction

*Trimma* Jordan & Seale, 1906 is among the most species-rich gobiid genera with 117 described species (Winterbottom 2019; Winterbottom et al. 2023; Peinemann et al. 2025; Chang et al. 2025; Harefa and Chen 2025; Fricke et al. 2026). Its members are small, substrate-associated pygmygobies, many with narrow microhabitat preferences. Ongoing surveys and taxonomic studies continue to uncover geographically restricted lineages, cryptic diversity, and entirely new species (Winterbottom et al. 2014, 2020).

The Red Sea, long recognized as an endemism hotspot, hosts a diverse and distinctive *Trimma* fauna (Golani and Fricke 2018; Bogorodsky and Goren 2023). Ten described species are currently known from the basin: *T. avidori* (Goren, 1978), *T. barralli* Winterbottom, 1995, *T. berumeni* Peinemann, Pombo-Ayora, Rich, Fox & Coker, 2025, *T. filamentosus* Winterbottom, 1995, *T. fishelsoni* Goren, 1985, *T. flavicaudatum* (Goren, 1982), *T. mendelssohni* (Goren, 1978), *T. nubarum* Winterbottom, Bogorodsky & Alpermann, 2023, *T. quadrimaculatum* Hoese, Bogorodsky & Mal, 2015, and *T. sheppardi* Winterbottom, 1984. Eight of these ten species appear endemic to the region. Notably, three of these (*T. quadrimaculatum*, *T. nubarum*, and *T. berumeni*) have been described since 2015. Furthermore, COI divergence indicates that *T. mendelssohni* may be restricted to the Red Sea and Socotra, while its southwestern Indian Ocean population could represent an undescribed species. Similarly, COI divergence within the widespread *T. sheppardi* suggests its Red Sea lineage may also represent a distinct, restricted species, though additional sampling and analyses are needed. Red Sea endemism in *Trimma* (80+%) is markedly higher than the overall proportion of endemic reef fishes in the Red Sea and Gulf of Aden (19.3%) and also exceeds the rate reported for Red Sea gobiids (~27%) more broadly (Bogorodsky and Randall 2019).

In May 2023, two of the authors (VNP and MDTT) collected two specimens of a distinctive *Trimma* in the northern Red Sea at a depth of 30 m. The species bears golden spotting on the caudal and dorsal fins and shows similarity to *Trimma anaima* Winterbottom, 2000, a widespread Indo-West Pacific species not reported from the Red Sea. Extensive subsequent surveys throughout the Red Sea did not yield additional material, consistent with either rarity or a deeper primary habitat. In Peinemann et al. (2025), this taxon was referred to as “*Trimma* sp. 1.” Here it is formally described as *Trimma charon* sp. nov. and diagnosed against congeners. In addition, we provide an identification key to all *Trimma* known from the Red Sea, accompanied by photographs of each species to facilitate reliable recognition in future surveys and collections.

## Material and methods

Morphometric measurements and meristic counts were taken according to standardized procedures developed by Richard Winterbottom to maintain continuity with prior *Trimma* studies (Winterbottom 2019; Winterbottom et al. 2023, and references therein). The order and wording of the diagnosis and description mirror the character sequence used in the genus-level key (Winterbottom 2019) and recent species accounts (Winterbottom et al. 2023). Cephalic sensory papillae are documented using established row nomenclature (Winterbottom 2011; Winterbottom et al. 2015). Measurements were made with digital calipers and on a ZEISS SteREO Discovery.V20 stereomicroscope operated via AxioVision. Characters in the description are of the holotype, with those of the paratype given in parentheses when different.

High-magnification images of preserved features were captured through the ZEISS SteREO Discovery.V20 and focus-stacked in AxioVision. Focus-stacked images were edited, aligned, and blended using Adobe Photoshop (version 26.10) and Adobe Lightroom Classic (version 14.5.1). Figure panels were assembled and edited in Adobe Illustrator (version 29.8.1).

Specimens were collected on SCUBA at a depth of 30 m using clove oil and hand nets. Live and freshly deceased coloration was documented immediately after capture and from *in situ* observations. Ex-situ images were taken in an upright photo tank with a Nikon Z7 camera body, Nikon Z MC 105 mm f/2.8 macro lens, and a Godox MF12 Macro flash. To improve visibility of diagnostic features, especially the cephalic sensory papillae and scales, specimens were briefly stained with cyanine blue 5R (acid blue 113) (Akihito et al. 1993; Saruwatari et al. 1997).

The dichotomous key to Red Sea *Trimma* species was developed using the key of Winterbottom (2019), original species descriptions and subsequent redescriptions, together with examination of specimens collected by the authors.

Type specimens have been deposited in the ichthyology collection of the Royal Ontario Museum (**ROM**). All fieldwork was conducted under approval 20IACUC05 issued by the KAUST Institutional Animal Care and Use Committee (**IACUC**).

## Results

### Trimma charon sp. nov.

Taxon classificationAnimaliaPerciformesGobiidae

BE556840-AE3F-50FA-91AA-5580F5DEE41F

https://zoobank.org/2527EEEE-5B09-4491-A165-F2DAE18BE838

Figs 1, 2, 4 F

Trimma sp. 1 – Peinemann et al. 2025: 86 (in the maximum-likelihood tree).

#### Common name.

Charon’s pygmygoby.

#### Type locality.

Saudi Arabia, Red Sea: Walih Island, 27.7897°N, 35.1630°E.

#### Type material.

***Holotype***. • ROMI118236, 16.8 mm SL, Red Sea, Saudi Arabia, Walih Island, 27.7897°N, 35.1630°E, outside cave of steep reef slope, 30 m depth, collected with clove oil and hand net, V.N. Peinemann, 15 May 2023. ***Paratype***. • ROMI118237, 12.1 mm SL, same data as for holotype.

#### Diagnosis.

A species of *Trimma* distinguished by the following combination of characters: predorsal midline with scales (present in larger specimen); fifth pelvic-fin ray unbranched and 41–43% length of fourth; cheek and opercle scaleless; pectoral-fin rays 14, all rays unbranched; dorsal fins VI + I,8; first dorsal fin rounded, second and third spines subequal; no elongate dorsal-fin spines; bony interorbital width 55–62% of pupil diameter; cephalic sensory papillae row c with 6 papillae. Live coloration translucent with a distinct, broad, orange-red midlateral stripe extending from eye to caudal-fin base and interrupted by three narrow pale bars; numerous golden spots on caudal fin extending onto caudal peduncle; iris divided into four uneven sectors (dorsal and ventral white; anterior and posterior orange, posterior smaller).

#### Description.

Based on holotype (16.8 mm SL) and one paratype (12.1 mm SL), the latter in parentheses if different. Dorsal fins VI + I,8, first fin rounded in shape, second and third spine longest (but not elongate) and approximately equal in length, extending to base of second dorsal-fin spine when adpressed; some second dorsal-fin rays branched; second dorsal fin reaching posteriorly 46% (38%) of caudal peduncle length; anal fin I,8, some rays branched; anal fin reaching posteriorly 51% (33%) of caudal peduncle length; pectoral-fin rays 14, all unbranched; pectoral-fin length 23.2% (24.1%) of SL, fin reaching posteriorly to above urogenital papilla; pelvic-fin I,5, fifth ray unbranched and 41% (43%) length of fourth ray, which reaches posteriorly to between base of first and second anal-fin ray, pelvic-fin rays 1 to 4 with one branching point each; membrane connecting fourth and third ray extending 60 to 80% length of fourth ray; basal membrane vestigial; no pelvic frenum; caudal fin with 2 dorsal and 2–3 ventral segmented unbranched rays, and 5 dorsal and 6 ventral segmented branched rays.

**Figure 1.**
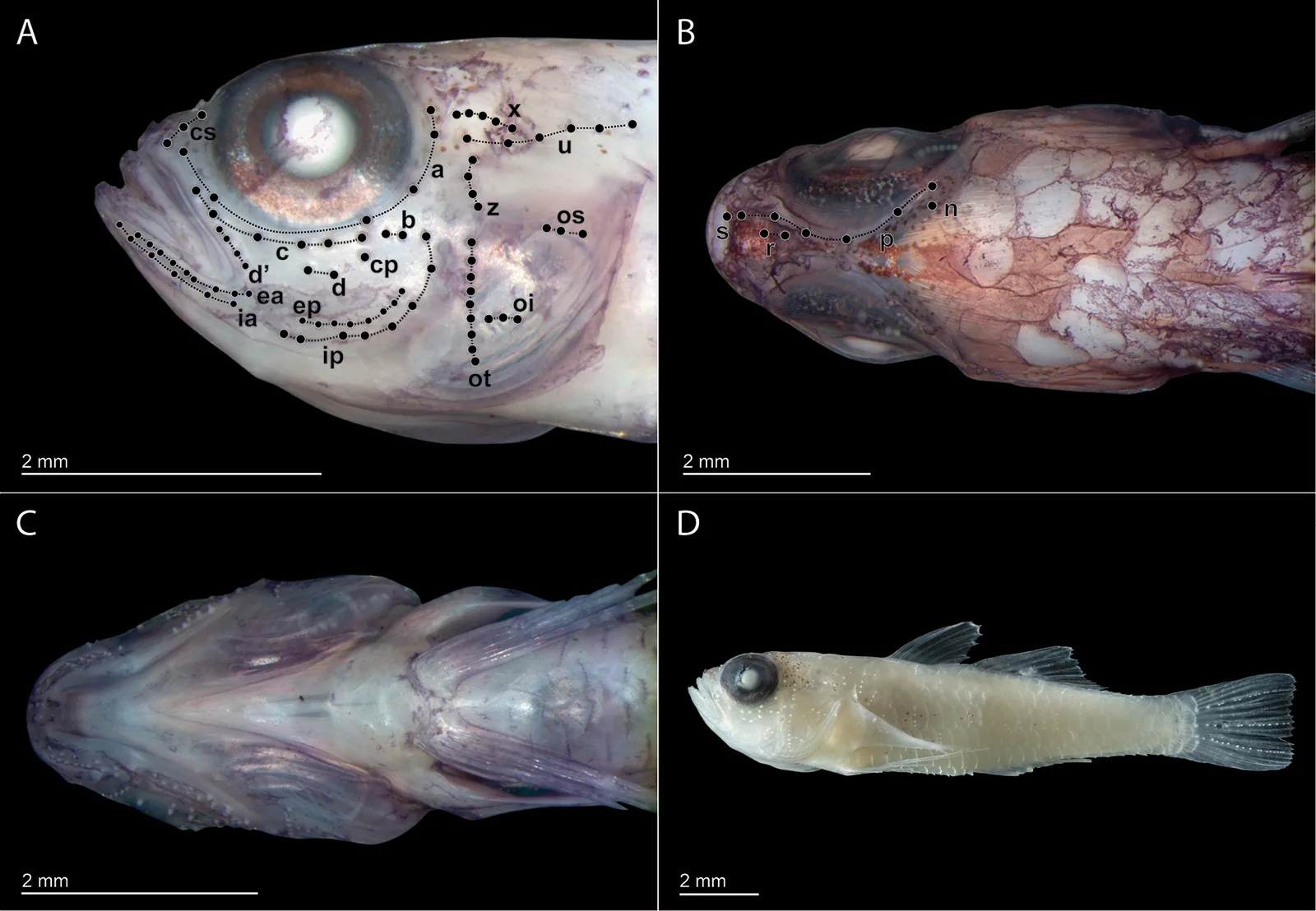
Cephalic sensory papillae and other diagnostic features of *Trimma charon* sp. nov. **A**. Lateral view of head (paratype), showing cephalic sensory papillae, stained with cyanine blue; **B**. Dorsal view of head (holotype), showing papillae rows across the interorbital region and scales in the predorsal midline, stained with cyanine blue; **C**. Ventral view of head (paratype), showing cephalic sensory papillae, gill opening and pelvic-fin bases, stained with cyanine blue; **D**. Lateral view of whole specimen (paratype). Panel (**B**) shows the holotype (16.8 mm SL); panels (**A, C, D**) show the paratype (12.1 mm SL); all specimens preserved in ethanol. Scale bars 2 mm.

Scales in longitudinal series 24; anterior transverse scales 8 (7), posterior transverse scales 7 (8); cheek and opercle scaleless; scales present on predorsal midline; three irregular rows of scales overlapping midline immediately anterior to dorsal-fin base, followed by a short unscaled interval (likely due to loss of deciduous scales) with space for 2–3 additional scales, and then a single median scale anteriorly (Fig. 1B); smaller specimen lacking predorsal scales, possibly due to abrasion or ontogeny; body scales ctenoid; scales on sides of nape, chest, and base of pectoral fin (including dorsally) cycloid; pectoral-fin base with two large vertically aligned scales; ventral midline anterior to pelvic-fin base with two scales in larger specimen, absent in smaller specimen; circumpeduncular scales 12; scale rows in ventral midline between base of last anal-fin ray and first procurrent caudal-fin ray 8.

Gill opening extending anteroventrally to between middle and posterior third of pupil (Fig. 1C); anterior naris tubular, about 1.5 times as long as wide, extending anteriorly to near anterior margin of the upper lip; posterior naris oval and pore-like with raised rim, separated from fleshy margin of orbit by approximately 1.5–2 times its diameter; bony interorbital width 55% (62%) of pupil diameter; posterior interorbital trough with sloping sides, extending posteroventrally to between dorsalmost papilla of row *a* and beginning of papilla row *u*; no dermal ridge on midline of nape extending anteriorly from origin of first dorsal fin.

Caudal peduncle depth as percentage of caudal peduncle length 48% (45%); head length 31% (28%) of SL; as percentage of head length: horizontal eye diameter 35% (41%); snout length 15% (16%); upper-jaw length 31% (33%).

Number of cephalic sensory papillae in each row (Fig. 1A, B): a = 6; b = 1 (2); c = 6; d = 3 (2); d‘ = 4; p = 6; e-anterior = 8 (9); e-posterior = 10 (8); i-anterior = 6; i-posterior = 7 (8); cs“ = 3; ot = 8 (9); os = 3; oi = 3; cp = 1; n = 1; x = 4 (5); u = 6; z = 3 (4); f = 2; r = 2; s = 1.

#### Color pattern, live (Fig. 2).

Body translucent with a distinct, broad, orange-red stripe extending from the eye to the base of the hypural plate, about one-third the body depth in width. This stripe is interrupted by three narrow pale bars, two positioned beneath the second dorsal fin and one on the caudal peduncle. Scale pockets along the stripe with darker orange-red pigmentation. Snout and upper jaw orange-red; anterior tubular naris blackish to dark red; lower jaw and cheek white to translucent. Iris divided into four uneven portions of alternating color; dorsal and ventral portions white and approximately equal in size; anterior and posterior portions orange, with the posterior portion smaller, about 50–60% the size of the anterior. Caudal fin with numerous golden spots, each one-third to one-half the pupil diameter in size; spots extend anteriorly onto the posterior caudal peduncle, forming three vertically aligned spots at the base of the caudal fin and another three at the posterior margin of the peduncle. Additional golden spots present on the ventral (1–2) and dorsal (up to 4) margins of the caudal peduncle. Remaining caudal fin hyaline, membranes with scattered white iridocytes. Dorsal fins hyaline with scattered white iridocytes (fewer in smaller individual) and diffuse golden spots. Anal fin hyaline with scattered white iridocytes and a diffuse golden stripe along the central 50–60% of the fin. Pelvic and pectoral fins hyaline and unpigmented.

**Figure 2.**
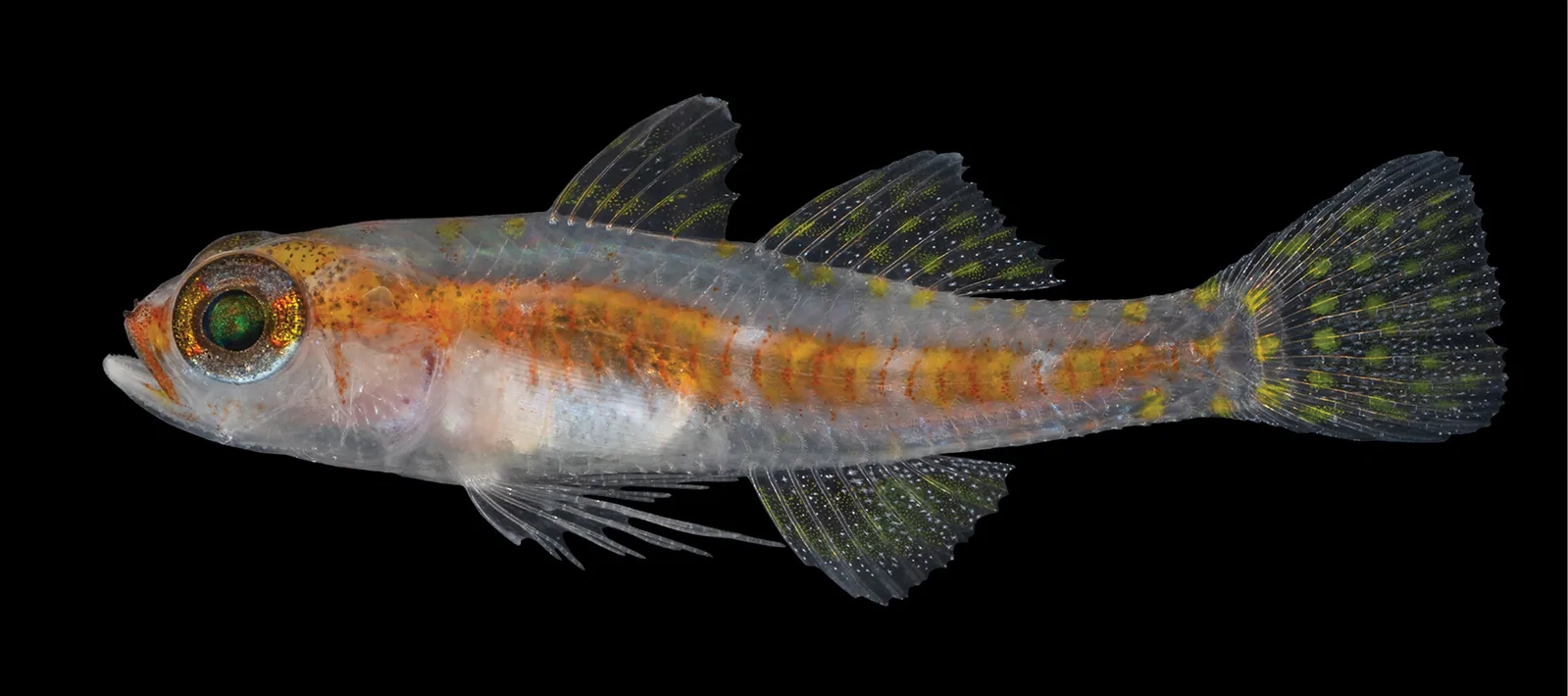
Freshly collected *Trimma charon* sp. nov., paratype, live, ROMI118237, 12.1 mm SL. Photograph: V.N. Peinemann.

**Figure 3.**
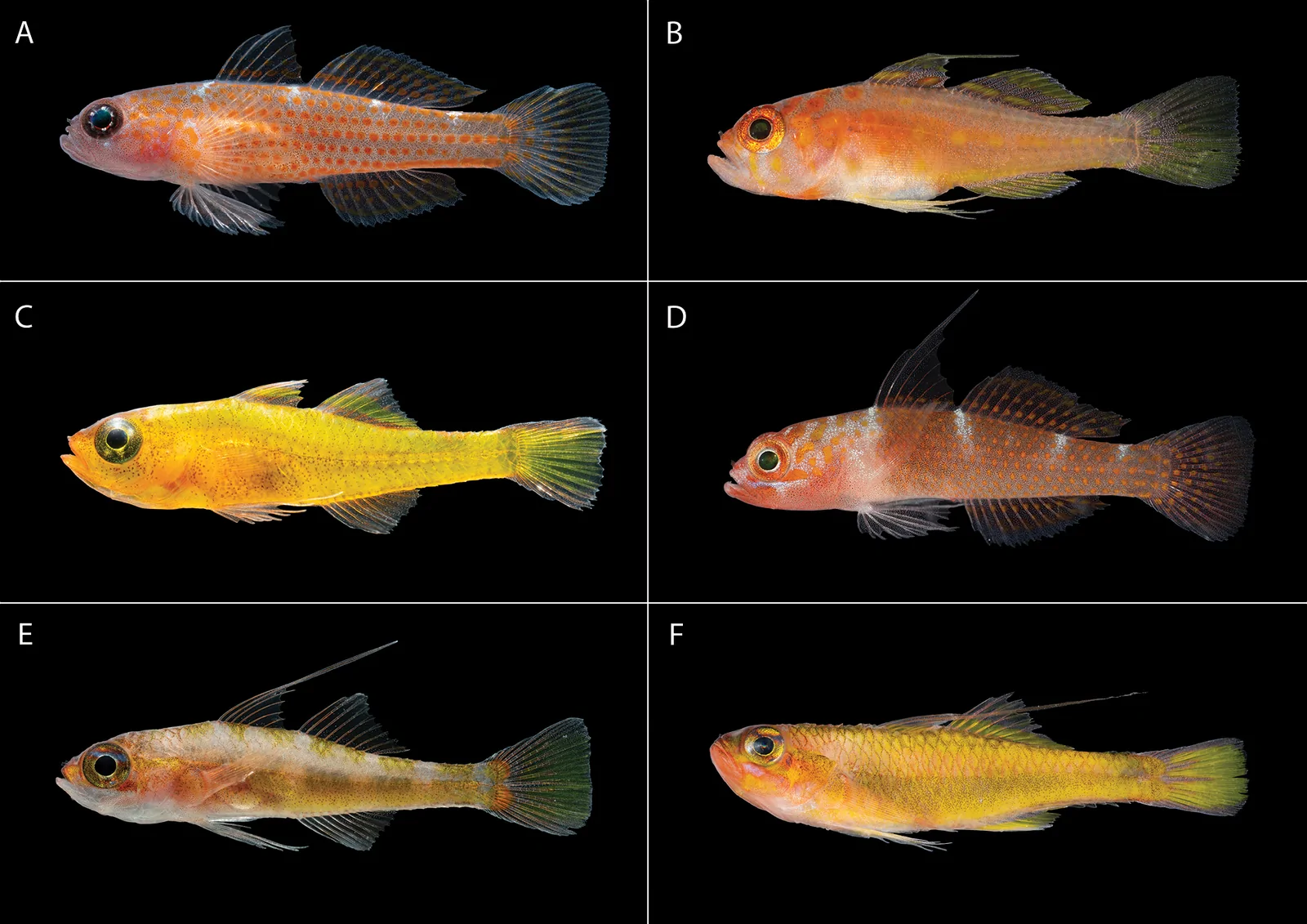
Species of *Trimma* recorded from the Red Sea (part 1; part 2, see Fig. 4). **A**. *T. avidori*; **B**. *T. barralli*; **C**. *T. berumeni*; **D**. *T. filamentosus*; **E, F**. *T. fishelsoni* (**E**. Specimen from Thuwal, central Red Sea; **F**. Specimen from Straits of Tiran, northern Red Sea / Gulf of Aqaba). All specimens collected and photographed in the Red Sea. Photographs by V.N. Peinemann.

**Figure 4.**
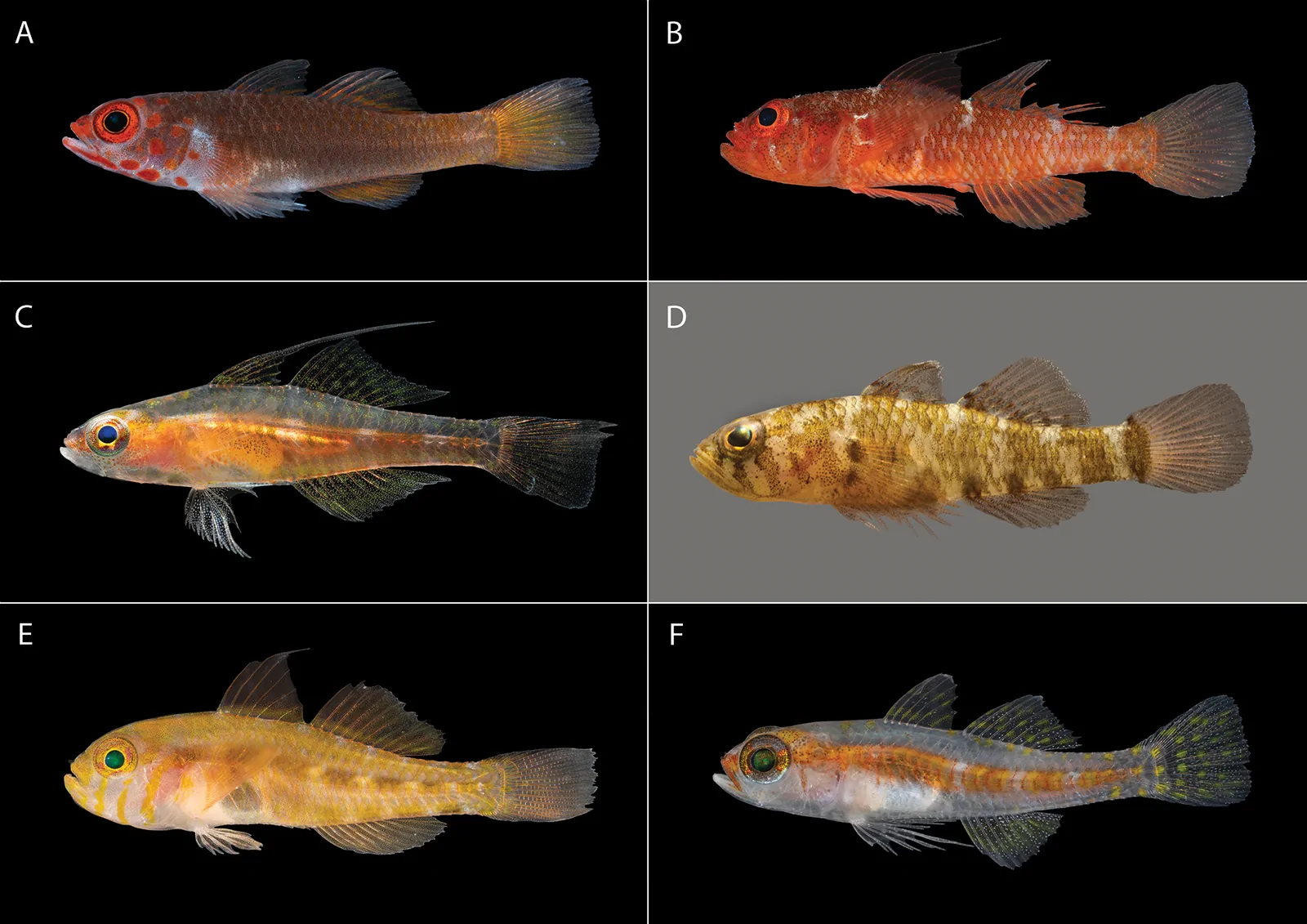
Species of *Trimma* recorded from the Red Sea (part 2). **A**. *T. flavicaudatum*; **B**. *T. mendelssohni*; **C**. *T. nubarum*; **D**. *T. quadrimaculatum*; **E**. *T. sheppardi*; **F**. *T. charon* sp. nov. All specimens collected and photographed in the Red Sea. Photographs by V.N. Peinemann, except *T. quadrimaculatum* (panel **D**) by S.V. Bogorodsky.

#### Color pattern, preserved (Fig. 1D).

Body pale white throughout. An amorphous small cluster of melanophores present posterodorsal to the eye. Sparse melanophores scattered along the midlateral region of the body in the position of the orange-red stripe seen in life. Fins hyaline, membranes of the dorsal, anal, and caudal fins with a few white iridocytes. Pectoral and pelvic fins hyaline without pigmentation. Iris mostly black in preservative, with faint silver-white visible in the anterior and posterior portions corresponding to the orange areas in life; dorsal and ventral portions fully black.

#### Etymology.

The species is named *Trimma charon* after Charon, the ferryman of the underworld in Greek mythology, who carried the souls of the dead across the river Styx in exchange for coins (Charon’s obols). The name refers to the distinctive golden spots on the caudal fin and peduncle, reminiscent of the coins. To be treated as a noun in apposition. The suggested common name is Charon’s pygmygoby.

#### Distribution and habitat.

The species is known only from two specimens collected at Walih Island in the northern Red Sea (27.7897°N, 35.1630°E). Both were observed at the bottom of a cave entrance at 30 m depth, resting on dead carbonate substrate. The site is a steep reef slope descending beyond 100 m, with a hard carbonate bottom that, unusually for the Red Sea, supported little structure and few living or recently dead corals. Despite extensive surveys of comparable habitats at similar depths across other Red Sea reefs, no additional specimens were found. The species may therefore be genuinely rare, or 30 m may represent the shallowest limit of its distribution, with greater abundance occurring at deeper depths. The apparent rarity of this species, or its restriction to deeper and northern habitats, is further supported by its absence from the extensive rotenone sampling stations of Coker et al. (2018) and Pombo-Ayora et al. (2024), as well as from the larval barcoding survey of Isari et al. (2017).

#### Comparisons.

The species most similar to *T. charon* is the Indo-West Pacific *T. anaima*. Both share a prominent orange-to-red midlateral stripe, all pectoral-fin rays unbranched, an unbranched fifth pelvic-fin ray, VI + I,8 dorsal-fin and I,8 anal-fin formulas, and no elongate dorsal-fin spines. *Trimma charon* differs from *T. anaima* by the presence of scales on the predorsal midline (scales absent in *T. anaima*; although also absent in the small paratype of *T. charon*) and pectoral-fin rays 14 (vs. 15–17, usually 16 rays). Live coloration readily separates the species: in *T. charon* the midlateral orange-red stripe is restricted to the central third of the body and interrupted by three narrow pale bars (vs. continuous and extending ventrally on the caudal peduncle in *T. anaima*); *T. charon* has distinct golden spots on the caudal and dorsal fins (absent in *T. anaima*, except sometimes along the dorsal and ventral margins of the caudal peduncle); and the iris of *T. charon* is divided into four uneven portions, with dorsal and ventral sectors white and anterior and posterior sectors orange, the posterior smaller (vs. iris uniformly bright red with a dorsomedial electric-blue stripe in *T. anaima*). Almost all body coloration of *T. charon* fades in preservative, but the four uneven iris portions remain faintly visible with subtle silver-white in the anterior and posterior portions (vs. uniform in *T. anaima*).

In the key to the genus *Trimma* by Winterbottom (2019), *T. charon* keys out to couplet 71, alongside *T. imaii* Suzuki & Senou, 2009 and *T. matsunoi* Suzuki, Sakaue & Senou, 2012. It differs from both species in having a bony interorbital width 55–62% of pupil diameter (vs. ~40% in *T. imaii* and ~75% in *T. matsunoi*) and in its live coloration, which includes a distinct midlateral orange-red stripe absent in both *T. imaii* and *T. matsunoi*. It can be further distinguished from *T. imaii* by having 8 dorsal-fin rays (vs. 9).

Within the Red Sea, *Trimma charon* is distinct from congeners. It bears some superficial resemblance to *Trimma nubarum* and *Trimma fishelsoni*, both of which combine orange-to-red body coloration with areas of transparency. *Trimma nubarum* additionally has faint yellow spots on the caudal and dorsal fins. However, both *T. nubarum* and *T. fishelsoni* possess an elongate filamentous second spine of the first dorsal fin, which is not elongate in *T. charon*.

##### Molecular sequences of type material

Sequences of the vouchers examined here were amplified, sequenced, and analyzed in Peinemann et al. (2025) and are made available here. GenBank accession numbers are provided in Table 1.

**Table 1.** GenBank accession numbers for the type specimens of *Trimma charon*. Voucher catalog numbers, accession numbers for each locus (COI, 16S, Ptr, S7I1), and GenSeq nomenclature (Chakrabarty et al. 2013) are given for the holotype and paratype. Sequences were originally generated by Peinemann et al. (2025).

**Species**	**Specimen catalog number**	**GenBank accession number**				**GenSeq nomenclature**
		**COI**	**16S**	**Ptr**	**S7I1**	
*Trimma charon*	ROMI118236	PZ538839	PZ538849	PZ548169	PZ548170	genseq-1 COI, 16S, Ptr, S7I1 (holotype)
*Trimma charon*	ROMI118237	PZ538840	NA	NA	NA	genseq-2 COI (paratype)

##### Key to *Trimma* species of the Red Sea

Habitat, depth, and relative abundance observations for Red Sea *Trimma* species, based on extensive field surveys by the authors, are provided in Table 2.

**Table 2.** Habitat and depth observations for *Trimma* species recorded from the Red Sea. Habitat and depth information is based on *in situ* observations and collections by the authors of this study during extensive SCUBA surveys along the Saudi Arabian Red Sea coast, primarily from the Gulf of Aqaba south to the Farasan Banks. Information from the southernmost Red Sea and the Gulf of Aden is comparably limited, but records from Djibouti are based on Winterbottom (1995). Surveys were largely restricted to depths between 0 and 30 meters; therefore, the absence of species from greater depths should not be inferred from these observations. The information is intended to provide qualitative ecological context and practical field guidance.

**Species**	**Habitat and distribution notes**	**Depth range**
*Trimma avidori*	One of the most abundant cryptobenthic species on many Red Sea reefs. Occurs on both inshore and midshelf reefs but is most common in sheltered inshore reefs and lagoons, resting on vertical carbonate surfaces and overhangs while feeding on plankton, often seen exposed. Present throughout the Red Sea, confirmed from the Gulf of Aqaba to Djibouti.	0–30 m; especially common at 5–20 m
*Trimma barralli*	Observed inside small caves and crevices on exposed offshore reefs. Uncommon at shallower limits of its range but likely more abundant in deeper habitats. Likely present throughout the Red Sea, confirmed from the Gulf of Aqaba to the Farasan Banks and Port Sudan.	~30 m and deeper
*Trimma berumeni*	Inhabits large caves of exposed offshore reefs, usually 80 cm to several meters inside the cave. Often associated with CCA-covered surfaces. Occurs singly or in small groups (3–10 individuals), moving along vertical substrate and cave roofs. Currently known only from the Farasan Banks, but the first author observed similar specimens inside a wreck at Yanbu, Saudi Arabia and a reef cave off Thuwal, Saudi Arabia.	15–30 m; most common at ~30 m and likely more abundant below this depth
*Trimma charon* sp. nov.	Known from two specimens collected at the bottom of a cave entrance. Individuals moved along horizontal dead carbonate substrate just outside the cave. These specimens were collected in the northern Red Sea.	30 m (based on limited material)
*Trimma filamentosus*	Generally rare on most reefs but can be common to abundant on sheltered inshore reefs and lagoons with high sedimentation. Typically rests on vertical reef structures and overhangs, often exposed, feeding on plankton. Known from the Gulf of Aqaba to the Farasan Banks.	0–30 m; most common at 3–25 m
*Trimma fishelsoni*	Occurs in caves and under overhangs on exposed offshore reefs, typically along cave roofs and vertical walls. Individuals may hover just above the substrate or shelter among small crevices in the cave structure. Present from the Gulf of Aqaba to the Farasan Banks.	20–47 m
*Trimma flavicaudatum*	One of the most abundant *Trimma* species throughout the Red Sea. Common on both inshore and offshore reefs, but nearly always within dark microhabitats such as small caves, crevices, shaded overhangs, and narrow recesses. Present throughout the Red Sea to the Gulf of Tadjoura, Djibouti.	2–30 m; likely extends deeper
*Trimma mendelssohni*	Moderately common, but not abundant, on exposed midshelf and offshore reefs. Typically rests on vertical surfaces of small caves, crevices, and overhangs in areas rich in crustose coralline algae (CCA). Highly cryptic and blends well with CCA-dominated substrate. Present throughout the Red Sea to Socotra, Yemen. Less than 2% COI divergence from Gulf of Aqaba to Socotra. Southwest Indian Ocean population likely represents an undescribed species.	0–20 m; most frequent from the shallow reef crest to ~10 m
*Trimma nubarum*	Occurs near large cave entrances and under substantial overhangs on exposed offshore reefs. Forms dense schools of 20–30+ individuals that hover in the shaded zone near these structures. Likely present throughout the Red Sea.	15–30 m; more common near ~30 m and presumably more common below this depth
*Trimma quadrimaculatum*	Relatively uncommon and shy. Typically found within small caves, crevices, and CCA-covered recesses of fringing and exposed seaward reefs. Most individuals are seen sheltering inside narrow reef-wall cavities. Although generally sparse, the species was observed to be locally common at one sheltered *Peyssonnelia*-dominated site off Thuwal (Mutarbej), where individuals occupied cave structures and the reef framework created by the red algae; this may represent a habitat where it can achieve higher local abundance, though this observation is based on a single site. Known from the northern to central Red Sea, from the Al Wajh Bank to Jeddah.	5–30 m; locally abundant at an offshore *Peyssonnelia*-dominated site at ~20–30 m
*Trimma sheppardi*	Typically observed near small crevices and cave entrances on exposed offshore reefs, usually resting on vertical or slightly overhanging carbonate surfaces. Uncommon in shallow water but presumably more abundant at greater depths. 3.7% COI divergence between Red Sea and Raja Ampat, Indonesia. Additional material and analyses needed to determine if Red Sea population may be an undescribed endemic species.	~30 m and deeper

**Table:** 

1	Predorsal midline scaleless	**2**
–	Predorsal midline with at least some scales, which may overlap from each side	**4**
2(1)	Posterior naris separated from margin of orbit; no interorbital and postorbital trenches; body with series of 7–8 black internal spots along vertebral column and a small black spot at upper end of gill opening, no small spots in longitudinal rows on body; iris with faint orange spot anteriorly and posteriorly and yellow inner ring	***T. sheppardi*** (Figs 4E, 5E)
–	Posterior naris attached (adnate) to margin of orbit; interorbital and postorbital trenches present; body with longitudinal rows of small spots; iris not as described above	**3**
3(2)	Second dorsal fin usually with 10 soft rays; second spine of first dorsal fin not elongate, reaching to third ray of second dorsal fin; body with longitudinal rows of small orange-red spots, those on head of similar size; iris with 6 small pale blue spots around inner margin; cheek without a pale stripe below eye	***T. avidori*** (Figs 3A, 5A)
–	Second dorsal fin usually with 11 soft rays; second spine of first dorsal fin elongate, reaching to or beyond middle rays of second dorsal fin; body with longitudinal rows of small orange-yellow spots, those on head twice as large; iris dark orange-red with white inner ring; cheek with a pale stripe below eye	***T. filamentosus*** (Figs 3D, 5B)
4(1)	Posterior naris attached (adnate) to margin of orbit; interorbital and postorbital trenches obvious; fifth pelvic-fin ray with many dichotomous branches; dorsal half of body with 6 narrow white bars; iris with two indistinct bluish bars dorsally and orange inner ring	***T. mendelssohni*** (Figs 4B, 6C, 6D)
–	Posterior naris separated from margin of orbit; interorbital and postorbital trenches absent or only interorbital trench developed; fifth pelvic-fin ray with 1–2 branch points or unbranched; color of body and iris not as described above	**5**
5(4)	Fifth pelvic-fin ray unbranched	**6**
–	Fifth pelvic-fin ray with 1–2 branch points	**8**
6(5)	Second spine of first dorsal fin elongate, filamentous, reaching posteriorly to posterior half of second dorsal fin or beyond when adpressed; pectoral-fin rays 15–17, middle rays branched or unbranched; bony interorbital width about equal to pupil diameter; iris with white cross-stripe above pupil	***T. fishelsoni*** (Figs 3E, 3F, 6G)
–	Second spine of first dorsal fin not elongate, reaching to base of second dorsal-fin spine when adpressed; pectoral-fin rays 14–15, all rays unbranched; bony interorbital width 55–84% of pupil diameter; iris without white stripe	**7**
7(6)	Second dorsal fin with 7 soft rays; bony interorbital width 81–84% of pupil diameter; cheek with 5 papillae in row c; body uniform yellow; median fins unspotted and dorsal fins with yellow-green longitudinal band running along central third; iris yellow, densely speckled with melanophores	***T. berumeni*** (Fig. 3C)
–	Second dorsal fin with 8 soft rays; bony interorbital width 55–62% of pupil diameter; cheek with 6 papillae in row c; body translucent with a broad orange-red midlateral stripe; median fins with small yellow spots; iris multicolor divided into uneven segments	***T. charon* sp. nov**. (Fig. 4F)
8(5)	Second spine of first dorsal fin elongate, filamentous, reaching posteriorly to posterior half of second dorsal fin or beyond when adpressed	**9**
–	Second spine of first dorsal fin not elongate, reaching to or slightly beyond base of second dorsal fin spine when adpressed	**10**
9(8)	Pectoral-fin rays 13; bony interorbital width greater than 80% of pupil diameter; interorbital with rounded fleshy ridge; body with an orange-red stripe narrowing posteriorly, head without spots; iris white dorsally	***T. nubarum*** (Figs 4C, 6E, 6F)
–	Pectoral-fin rays 17–19, usually 18; bony interorbital width 50% or less of pupil diameter; interorbital trench developed; predorsal area with red spots; iris with 3 short white bars on dorsal margin, inner ring white	***T. barralli*** (Figs 3B, 5C, 5D)
10(8)	Pectoral-fin rays unbranched; no elongate papillae on nape; side of head and nape with red spots and streaks; iris dark red with white dorsal arc and white inner ring	***T. flavicaudatum*** (Figs 4A, 6A, 6B)
–	Most pectoral-fin rays unbranched, some branched; nape with pair of elongate papillae; head and body brown with gray and white mottling, with dark brown spots at anal-fin base and ventrally on caudal peduncle; iris dark golden-brown with yellow inner ring	***T. quadrimaculatum*** (Figs 4D, 5F)

## Discussion

With the description of *T. charon*, the number of described *Trimma* species recorded from the Red Sea increases from 10 to 11, at least nine of which appear to be regionally endemic. This makes *Trimma* the most species-rich gobiid genus in the Red Sea, although genera such as *Gobiodon*, *Eviota*, and *Hetereleotris* are close in diversity and could surpass it as additional species are described. In contrast, *Trimma* diversity drops sharply outside the Red Sea and Gulf of Aden. Only two species, *T. winterbottomi* Randall & Downing in Randall et al. 1994 and *T. omanense* Winterbottom, 2000, are reported from Oman (Randall 1995; Al-Jufaili et al. 2010). Four species are currently known from Socotra, namely *T. flammeum* (Smith, 1959), *T. corallinum* (Smith, 1959), *T. mendelssohni*, and *T. winterbottomi*, highlighting a modest but distinct *Trimma* fauna at the southern margin of the region (Zajonz and Bogorodsky, unpublished). Coral reef development along most of the southern Omani and eastern Somali coasts is limited by persistent upwelling (Carbone and Accordi 2000; Vic et al. 2017; Junior 2022), likely preventing the establishment of various species of *Trimma*. This geographic and ecological barrier isolates Red Sea reefs from the nearest comparable habitats in the western Indian Ocean and probably contributes to the high rate of endemism observed in small-bodied, short-lived, and site-attached gobies such as *Trimma*.

**Figure 5.**
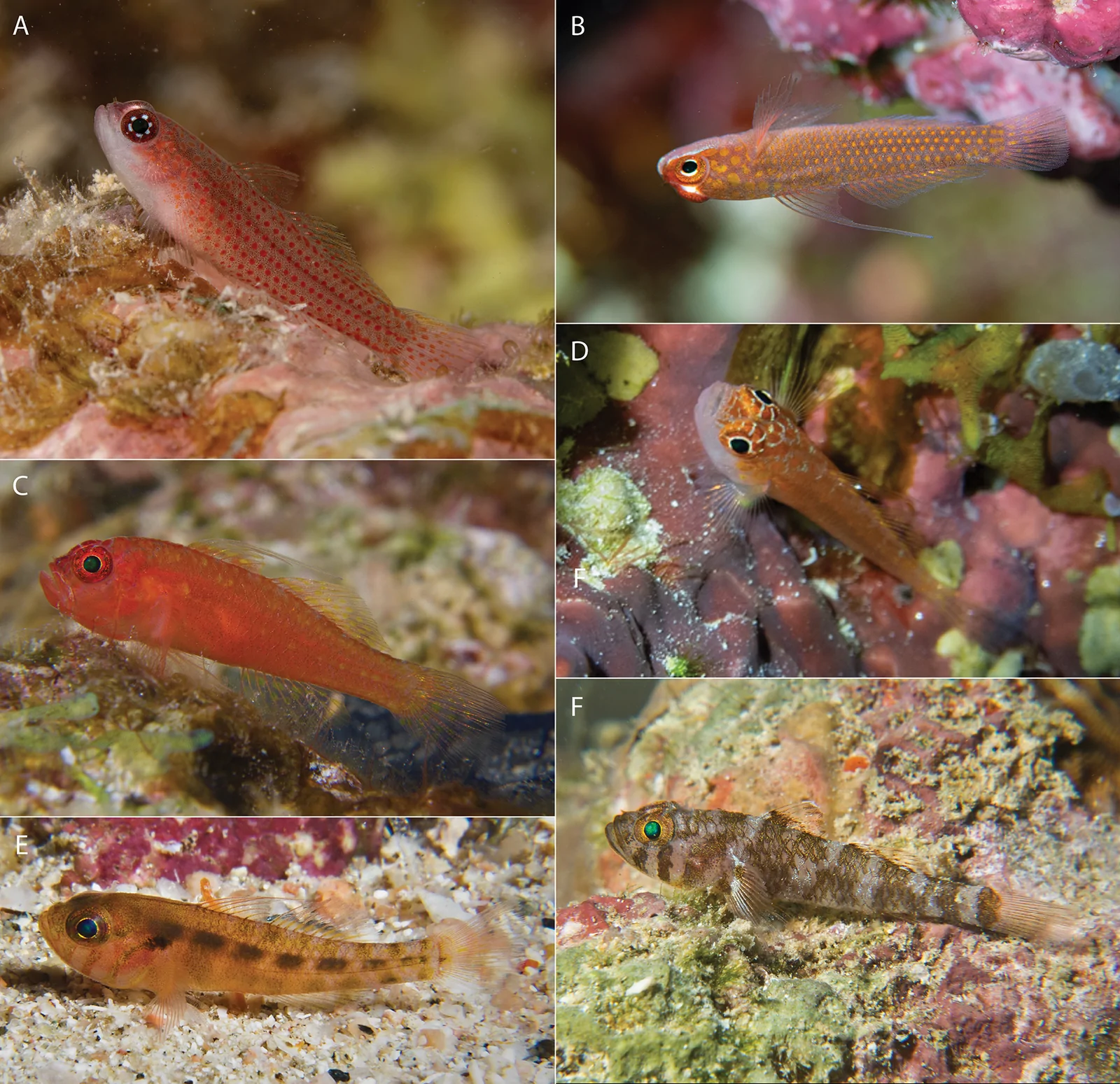
*In situ* photographs of Red Sea *Trimma* species (part 1; part 2, see Fig. 6). **A**. *T. avidori*; **B**. *T. filamentosus*; **C, D**. *T. barralli*; **E**. *T. sheppardi*; **F**. *T. quadrimaculatum*. All photographs taken in the Red Sea. Photographs by V.N. Peinemann (panels **A, D**); by F. Krasovec (panel **B**); by S.V. Bogorodsky (panels **C, E, F**).

**Figure 6.**
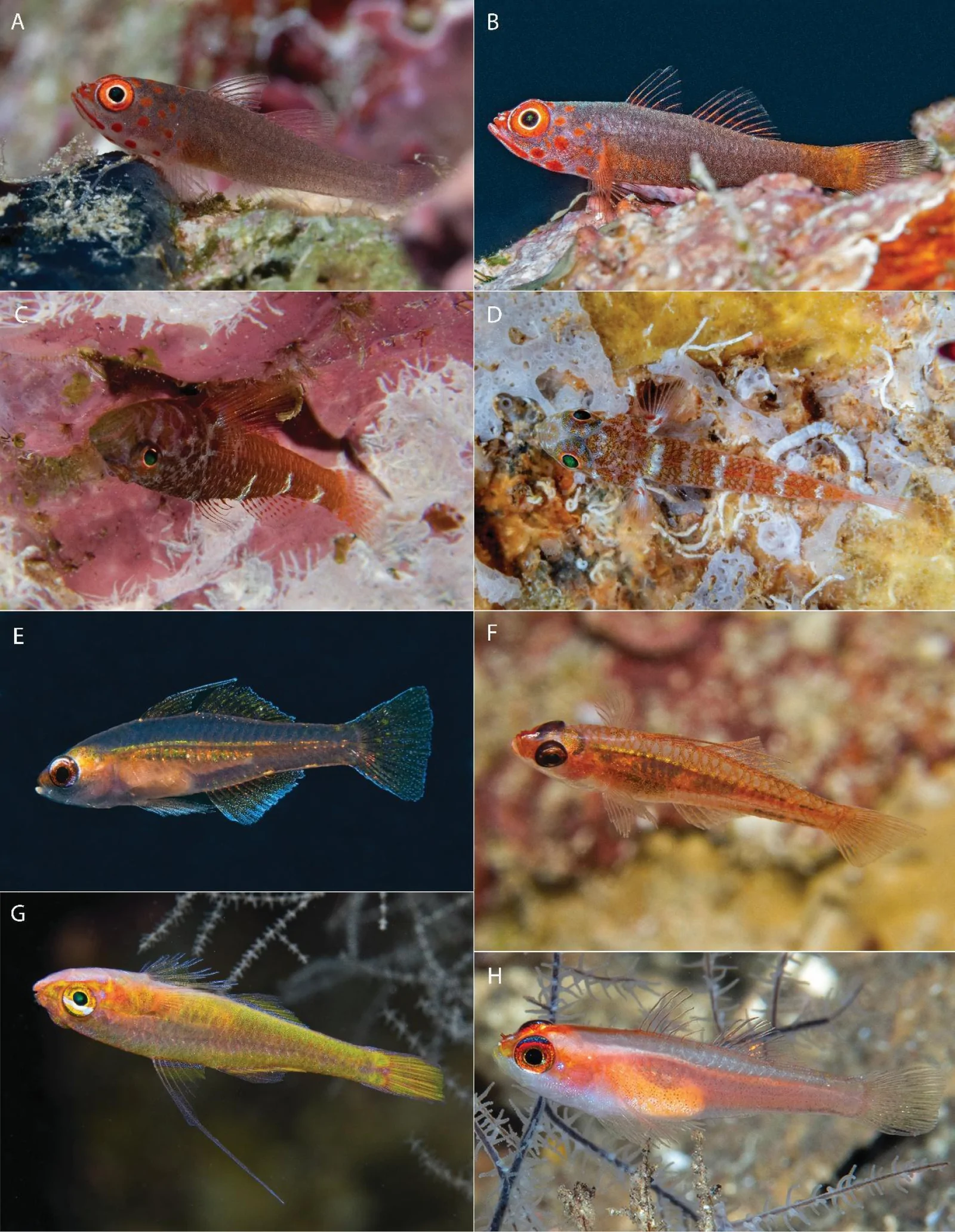
*In situ* photographs of Red Sea *Trimma* species (part 2) and *Trimma anaima*. **A, B**. *T. flavicaudatum*; **C, D**. *T. mendelssohni*; **E, F**. *T. nubarum*; **G**. *T. fishelsoni*; **H**. *T. anaima*. All photographs taken in the Red Sea except panel (**H**) (*T. anaima*; not reported in Red Sea, but similar to *T. charon*), photographed in Bali, Indonesia. Photographs by V.N. Peinemann (panels **A, D**); by S.V. Bogorodsky (panels **B, C, E, F, G**); by A. Ryanskiy (panel **H**).

Although broader phylogenetic analyses are needed to resolve the sister species of *T. charon*, it is morphologically most similar to *T. anaima*. Based on available COI sequences for 93 described species of *Trimma*, *T. anaima* is also genetically closest to *T. charon*, with a pairwise identity of 85–86% (Peinemann et al. 2025). Given that the Red Sea has supported modern coral reef habitats for only about 5.5 million years (Pensa et al. 2025), this would imply a COI divergence rate of at least ~3% per million years if *T. charon* is indeed endemic to the Red Sea and sister to *T. anaima*. This is well within substitution rates estimated for other small, short-lived reef gobies (Tornabene et al. 2015), but would nonetheless imply a prolonged presence in the Red Sea. An alternative possibility is that *T. charon* may be more widespread or sister to an unsampled or now-extinct lineage from the Arabian Peninsula or nearby regions. Notably, *T. anaima* is one of the most widely distributed *Trimma*, ranging from the Comoros (its type locality) to Fiji. In a COI analysis of 87 species, Winterbottom et al. (2020) found *T. anaima* to be among the few with a well-sampled single haplogroup spanning much of the Indo-Pacific, showing only ~1.5% COI divergence between populations from the Seychelles and the Philippines.

*Trimma charon* is currently known from only two specimens collected at a depth of 30 m on a reef with a steep slope descending beyond 100 m. To our knowledge, it has not been recorded in shallower surveys elsewhere in the Red Sea, suggesting that the species is either genuinely rare or more common on deeper mesophotic reefs, with our collection representing the shallower limit of its distribution. Cryptobenthic fishes remain poorly characterized in the mesophotic reefs of the Red Sea, as the majority of regional collections are restricted to depths shallower than 30 m. With *Trimma berumeni*, *T. charon* is the second new species of *Trimma* discovered from the Red Sea at 30 m within the last five years, and it is likely that additional species remain to be discovered at this depth and deeper into the mesophotic. Mesophotic collections continue to reveal new *Trimma* species throughout the Indo-Pacific (e.g., Winterbottom and Erdmann 2018; Winterbottom and Pyle 2022a, 2022b), and the Red Sea and wider Indian Ocean remain particularly undersampled at these depths.

## Supplementary Material

XML Treatment for Trimma charon

## Additional information

### Artificial Intelligence (AI) use

The authors accept full responsibility for the content of the manuscript, including the disclosure of any use of AI.

Regarding the use of AI in the preparation of this manuscript, the authors declare the following: Claude.

**Used for**: Language, style and writing.

Minor grammar polish and formatting check.

### Author ORCIDs

V.N. Peinemann https://orcid.org/0000-0002-7060-397X

S.V. Bogorodsky https://orcid.org/0000-0002-8679-9735

M.D. Tietbohl Talas https://orcid.org/0000-0002-1101-8412

M.L. Berumen https://orcid.org/0000-0003-2463-2742

### Data availability

All of the data that support the findings of this study are available in the main text.

## References

[B1] Akihito, Sakamoto K, Iwata A, Ikeda Y (1993) Cephalic sensory organs of the gobioid fishes. In: Nakabo T (Ed.), Fishes of Japan with Pictorial Keys to the Species. Tokai University Press, Tokyo, 1088–1116.

[B2] Al-Jufaili S, Hermosa G, Al-Shuaily S, Al-Mujaini A (2010) Oman Fish Biodiversity. Journal of King Abdulaziz University-Marine Sciences 21: 3–51. 10.4197/Mar.21-1.1

[B3] Bogorodsky SV, Goren M (2023) An updated checklist of the Red Sea gobioid species (Teleostei: Gobiiformes), with four new records. Zoology in the Middle East 69: 123–163. 10.1080/09397140.2023.2203978

[B4] Bogorodsky SV, Randall JE (2019) Endemic Fishes of the Red Sea. In: Rasul NMA, Stewart ICF (Eds) Oceanographic and Biological Aspects of the Red Sea. Springer Oceanography. Springer International Publishing, Cham, 239–265. 10.1007/978-3-319-99417-8_14

[B5] Carbone F, Accordi G (2000) The Indian Ocean Coast of Somalia. Marine Pollution Bulletin 41: 141–159. 10.1016/S0025-326X(00)00107-7

[B6] Chakrabarty P, Warren M, Page L, Baldwin CC (2013) GenSeq: An updated nomenclature and ranking for genetic sequences from type and non-type sources. ZooKeys 346: 29–41. 10.3897/zookeys.346.5753 PMC3821064 24223486

[B7] Chang C-W, Shen K-N, Chen I-S (2025) A new species of dwarf goby of *Trimma* (Teleostei: Gobiidae) from Taiping Island, South China Sea. Zootaxa 5738: 8–12. 10.11646/zootaxa.5738.1.4 42407840

[B8] Coker DJ, DiBattista JD, Sinclair-Taylor TH, Berumen ML (2018) Spatial patterns of cryptobenthic coral-reef fishes in the Red Sea. Coral Reefs 37: 193–199. 10.1007/s00338-017-1647-9

[B9] Fricke R, Eschmeyer WN, van der Laan R (2026) Eschmeyer’s Catalog of Fishes [electronic version, 02 February 2026]. California Academy of Sciences, San Francisco. http://research.calacademy.org/research/Ichthyology/Catalog/fishcatmain.asp

[B10] Golani D, Fricke R (2018) Checklist of the Red Sea Fishes with delineation of the Gulf of Suez, Gulf of Aqaba, endemism and Lessepsian migrants. Zootaxa 4509: 1–215. 10.11646/zootaxa.4509.1.1 30485948

[B11] Goren M (1978) A new gobiid genus and seven new species from Sinai coasts (Pisces: Gobiidae). Senckenbergiana Biologica 59: 191–203.

[B12] Goren M (1982) *Quisquilius flavicaudatus*, a new gobioid fish from the coral reefs of the Red Sea. Zoologische Mededelingen 56: 139–142.

[B13] Goren M (1985) *Trimma fishelsoni*, a new gobiid fish from the Gulf of Elat, northern Red Sea. Israel Journal of Zoology 33: 63–67.

[B14] Harefa T, Chen I-S (2025) *Trimma novemstriatum*, a new species of marine goby fish (Teleostei: Gobiidae) from Taiwan. Zootaxa 5738: 62–72. 10.11646/zootaxa.5738.1.11 42407847

[B15] Hoese DF, Bogorodsky SV, Mal AO (2015) Description of a new species of *Trimma* (Perciformes: Gobiidae) from the Red Sea, with a discussion of the generic separation of *Trimma* and *Priolepis*, with discussion of sensory papillae terminology. Zootaxa 4027: 538–550. 10.11646/zootaxa.4027.4.4 26624195

[B16] Isari S, Pearman JK, Casas L, Michell CT, Curdia J, Berumen ML, Irigoien X (2017) Exploring the larval fish community of the central Red Sea with an integrated morphological and molecular approach. PLOS ONE 12: e0182503. 10.1371/journal.pone.0182503 PMC5542619 28771590

[B17] Jordan DS, Seale A (1906) The fishes of Samoa: description of the species found in the archipelago, with a provisional check list of the fishes of Oceania. Bulletin of the Bureau of Fisheries 25: 173–455. 10.5962/bhl.title.46247

[B18] Junior OC (2022) Upwellings along the Coasts of Somalia and Arabia. London Journal of Science: Natural and Formal 22: 29–38.

[B19] Peinemann VN, Pombo-Ayora L, Rich WA, Fox MD, Coker DJ (2025) A new cave-dwelling species of *Trimma* (Teleostei, Gobiidae) from the Red Sea, with notes on Red Sea endemism in *Trimma* spp. ZooKeys 1255: 77–93. 10.3897/zookeys.1255.159341 PMC12531819 41113182

[B20] Pensa T, Baby G, Teillet T, Delaunay A, Delgado Huertas A, Afifi A (2025) Evolution of carbonate platforms in the northeast Red Sea during the last 23 million years. Scientific Reports 15: 10332. 10.1038/s41598-025-92219-x PMC11937587 40133459

[B21] Pombo-Ayora L, Nunes Peinemann V, Coker DJ, Berumen ML (2024) Length-Weight Relationships for 32 Species of Cryptobenthic Reef Fishes from the Red Sea. Journal of Applied Ichthyology 2024: 1454131. 10.1155/2024/1454131

[B22] Randall JE (1995) Coastal Fishes of Oman. University of Hawaii Press, xvii + 439 pp.

[B23] Randall JE, Downing N, McCarthy L, Stanaland BE, Tarr AB (1994) Fifty-one new records of fishes from the Arabian Gulf. Fauna of Saudi Arabia 14: 220–258.

[B24] Saruwatari T, López JA, Pietsch TW (1997) Cyanine Blue: a versatile and harmless stain for specimen observation. Copeia 1997: 840–841. 10.2307/1447302

[B25] Smith JLB (1959) Gobioid fishes of the families Gobiidae, Periophthalmidae, Trypauchenidae, Taenioididae, and Kraemeriidae of the Western Indian Ocean. Ichthyological Bulletin, Department of Ichthyology, Rhodes University 13: 185–225.

[B26] Suzuki T, Senou H (2009) Two new species of the genus *Trimma* (Perciformes: Gobiidae) from Japan and Palau. Bulletin of the Kanagawa Prefectural Museum (Natural Science) 38: 107–112.

[B27] Suzuki T, Sakaue J, Senou H (2012) Two new species of the gobiid fish genus *Trimma* (Actinopterygii: Perciformes: Gobiidae) from Japan and Palau. Bulletin of the National Museum of Nature and Science Series A 38: 67–77.

[B28] Tornabene L, Valdez S, Erdmann MV, Pezold F (2015) Support for a ‘Center of Origin’ in the Coral Triangle: cryptic diversity, recent speciation, and local endemism in a diverse lineage of reef fishes (Gobiidae: *Eviota*). Molecular Phylogenetics and Evolution 82: 200–210. 10.1016/j.ympev.2014.09.012 25300452

[B29] Vic C, Capet X, Roullet G, Carton X (2017) Western boundary upwelling dynamics off Oman. Ocean Dynamics 67: 585–595. 10.1007/s10236-017-1044-5

[B30] Winterbottom R (1984) A review of the gobiid fish genus *Trimma* from the Chagos Archipelago, central Indian Ocean, with the description of seven new species. Canadian Journal of Zoology 62: 695–715. 10.1139/z84-101

[B31] Winterbottom R (1995) Red Sea gobiid fishes of the genus *Trimma*, with the description of two new species. Revue Française d’Aquariologie 22: 93–98.

[B32] Winterbottom R (2000) Four new species of *Trimma* (Gobiidae), from the Indian and Western Pacific Oceans. aqua, Journal of Ichthyology and Aquatic Biology 4: 57–66.

[B33] Winterbottom R (2011) Six new species of the genus *Trimma* (Percomorpha; Gobiidae) from the Raja Ampat Islands, Indonesia, with notes on cephalic sensory papillae nomenclature. aqua, International Journal of Ichthyology 17: 127–163.

[B34] Winterbottom R (2019) An illustrated key to the described valid species of *Trimma* (Teleostei: Gobiidae). Journal of the Ocean Science Foundation 34: 1–61. 10.5281/zenodo.3525430

[B35] Winterbottom R, Erdmann MV (2018) Two new species of blue-eyed *Trimma* (Pisces; Gobiidae) from New Guinea. Zootaxa 4444: 471–483. 10.11646/zootaxa.4444.4.7 30313918

[B36] Winterbottom R, Pyle RL (2022a) A new species of *Trimma* (Pisces: Gobiidae) from the deep reefs of Palau, western Pacific Ocean. Zootaxa 5094: 595–600. 10.11646/zootaxa.5094.4.5 35391433

[B37] Winterbottom R, Pyle RL (2022b) A new species of *Trimma* (Teleostei: Gobiidae) from the deep reefs of Vanuatu, western Pacific Ocean. Journal of the Ocean Science Foundation 39: 2–8. 10.5281/zenodo.6456585

[B38] Winterbottom R, Hanner RH, Burridge M, Zur M (2014) A cornucopia of cryptic species - a DNA barcode analysis of the gobiid fish genus *Trimma* (Percomorpha, Gobiiformes). ZooKeys 381: 79–111. 10.3897/zookeys.381.6445 PMC3950426 24624015

[B39] Winterbottom R, Erdmann MV, Cahyani NKD (2015) New species of *Trimma* (Actinopterygii, Gobiidae) from Indonesia, with comments on head papillae nomenclature. Zootaxa 3973: 201–226. 10.11646/zootaxa.3973.2.1 26249856

[B40] Winterbottom R, Burridge M, Erdmann MV, Hanner RH, Zur M, Steinke C, Choffe K (2020) The cryptic cornucopia revisited–an extended analysis of the COI gene in the gobiid fish genus *Trimma* (Percomorpha: Gobiiformes). Journal of the Ocean Science Foundation 36: 91–132. 10.5281/zenodo.4403739

[B41] Winterbottom R, Bogorodsky SV, Alpermann TJ (2023) A new species of *Trimma* of the *T. taylori* species group (Teleostei: Gobiidae) from the Red Sea, Indian Ocean. Zootaxa 5353: 250–264. 10.11646/zootaxa.5353.3.3 38220686

